# Effect of electroacupuncture on restless legs syndrome (RLS) in hemodialysis patients: A protocol for systematic review and meta-analysis

**DOI:** 10.1097/MD.0000000000023629

**Published:** 2020-12-11

**Authors:** Jia-Ming Chen, Ping-Fang Chiu, Yu-Jun Chang, Po-Chi Hsu, Chia-Chu Chang, Lun-Chien Lo

**Affiliations:** a Graduate Institute of Chinese Medicine, China Medical University, Taichung; b Department of Traditional Chinese Medicine, Changhua Christian Hospital, Changhua; c Nephrology Division, Department of Internal Medicine, Changhua Christian Hospital, Changhua; d Epidemiology and Biostatistics Center, Changhua Christian Hospital, Changhua; e Division of Nephrology, Department of Internal Medicine, Kuang Tien General Hospital, Taichung; f School of Chinese Medicine, China Medical University, Taichung; g Department of Chinese Medicine, China Medical University Hospital, Taichung, Taiwan.

**Keywords:** acupuncture, heart rate variability, hemodialysis, protocol, restless leg syndrome

## Abstract

**Background:**

Restless legs syndrome (RLS) is frequent in dialysis patients and occurs predominantly in its most severe forms. The aim of the present study was to evaluate the effects of electroacupuncture (EA) in hemodialysis patients with RLS by heart rate variability (HRV) monitor.

**Methods:**

One hundred twelve subjects who were hemodialysis patients with RLS will be divided into 2 groups: experimental and control. Each subject will receive the treatment relevant to their group 2 times a week for 4 weeks. After 4 weeks of treatment the subject will enter a 2-week washout period, after which the subjects will switch groups. Measurements will include HRV recordings, International Restless Legs Syndrome Rating Scale (IRLSRS) and Insomnia Severity Index (ISI).

**Result:**

The results of this study will systematically evaluate the effectiveness and safety of electoracupuncture intervention for hemodialysis patients with RLS.

**Discussion:**

This study is the first investigation to analyze the relationship between EA and the change of HRV by an objective monitor. If the findings of the current trial are positive, this study will also help support an effective, safe and cheap approach to clinical treatment of this challenging disorder, help foster improved understanding *the relationship between* autonomic nervous system and RLS, and ultimately contribute to elucidate the mechanisms of EA.

**Trial registration:**

ClinicalTrials.gov Identifier: NCT04356794; registration date: April 22, 2020.

## 1 Introduction

### 1.1 Background and rationale

Restless legs syndrome (RLS), also known as Willis-Ekbom disease is a neurological sensory disorder with symptoms involving an often unpleasant or irresistible urge to move the legs that occurs during periods of inactivity, particularly in the evenings, and is transiently relieved by movement.^[1]^ A large population-based study in Europe and the United States found that 37% of patients with RLS symptoms had moderate to severe disease.^[2]^

RLS can be primary or can occur as a secondary disorder in association with medical conditions, including low iron levels, kidney failure, Parkinson disease, diabetes mellitus, rheumatoid arthritis, and pregnancy.^[3]^ Studies in patients with dialysis showed a prevalence of RLS ranging from 12% to 62%, which is significantly higher compared to the general population.^[4–8]^ A previous study concluded that the high prevalence of RLS among dialysis patients and the associations between the severity of RLS and the risk of new cardiovascular events and higher short-term mortality.^[9]^

Heart rate is modulated through effects of sympathetic and parasympathetic nervous systems, and analysis of changes in heart rate over time provides information about autonomic function.^[10]^ An important non-invasive method used widely to study these changes is heart rate variability (HRV). A previous study demonstrated that heart rate variability is related to RLS.^[11]^ A recent study indicated that a decrease in HRV is typically found in dialysis patients with abnormal HRV being considered an independent risk factor for mortality.^[12]^

Several previous studies have showed a relationship between acupuncture and heart rate variability in RLS. Yamamoto et al reported that standard acupuncture might improve the abnormal leg activity in RLS patients.^[13]^ Koo et al reported that electroacupuncture (EA) significantly increased in the LF/HF ratio in healthy volunteers.^[14]^ To our best knowledge, the HRV analysis on the therapeutic effect of EA in hemodialysis patients with RLS has not been performed earlier.

For these reasons, we will collaborate with a hemodialysis room in order to investigate HRV evaluation of EA treatment on RLS in hemodialysis patients. In this study, we decided to conduct a patient-assessor-blinded, randomized, sham controlled crossover pilot trial.

## 2 Objectives

The aim of this study is to assess the effectiveness of EA in the treatment of RLS. We evaluated the effects of EA in hemodialysis patients with RLS by HRV recordings, IRLSRS, and ISI. All adverse events will also be assessed.

### 2.1 Trial design

This study is a randomized-controlled crossover trial with a 2-week washout period between each crossover phase.

### 2.2 Methods: Participants, interventions and outcomes

#### 2.2.1 Study setting

**Figure 1:**
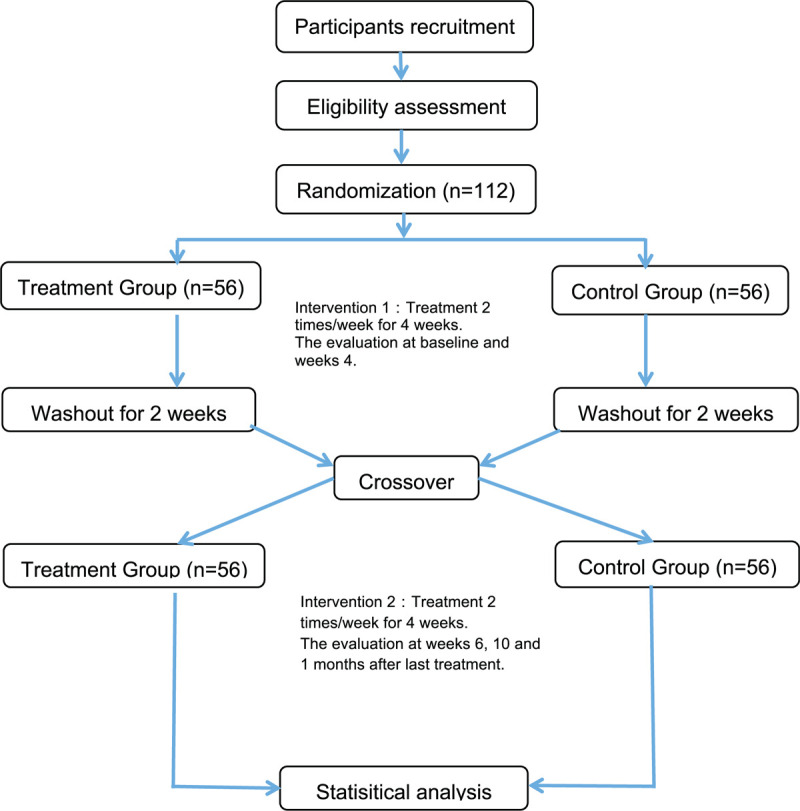
Flowchart of the study design.

The trials will be conducted at the Changhua Christian Hospital. This study will adhere to the recommendations of the Consolidated Standards of Reporting Trials (CONSORT)^[15]^ to allow for greater completeness, transparency and accuracy of reporting. The protocol for this study has been registered in the Clinical Trials register (ClinicalTrials.gov Identifier: NCT04356794). A flow diagram of the trial is shown in Fig. 1.

#### 2.2.2 Eligibility criteria

Subjects who voluntarily sign a consent form will undergo the trial according to the study design. When a subject is determined to be fit for participation based on inclusion and exclusion criteria, the subject will be randomly assigned to 1 of 2 groups in a ratio of 1:1. One group is the true EA experimental group and the other is the sham EA control group. Each group will receive their treatment 2 times a week for 4 weeks. After 4 weeks of treatment and a 2-week washout period, the control group will switch to the experimental group while the experimental group will switch to the control group. The subjects then undergo the treatment of their new group for another 4 weeks.

Treatment and assessment will be performed independently and the practitioners will not be involved in assessing the outcome of the treatment. The subjects, the outcome assessors and the statistician performing the data analyses will be blinded to the treatment allocation throughout the study.

#### 2.2.3 Ethics

This research protocol adheres to the principles of the Declaration of Helsinki and has been approved by the institutional review boards of the Changhua Christian Hospital (No.: 170217). Informed consent will be obtained from each participant before any treatment is given. All subjects will have the right to withdraw from the study at any time.

#### 2.2.4 Inclusion criteria

The inclusion criteria were as follows:

1. Informed consent to participate in the study;
2. Between the ages of 20 to 80 years;
3. A mean frequency of RLS symptoms during the last 6 months of more than twice per week;
4. A score of at least 20 on the International Restless Legs Syndrome Rating Scale (corresponding to severe RLS, IRLSRS);
5. A history of chronic renal failure treated with regular and continuous hemodialysis for at least 3 months;
6. No acupuncture treatment in the last 1 month.

#### 2.2.5 Exclusion criteria

The exclusion criteria were as follows:

1. An unwillingness to continue to participate in the study;
2. Vascular access in the leg area, such as an arteriovenous shunt;
3. The presence of peripheral neuropathy and vascular problems in the lower extremities;
4. Currently taking drugs for RLS, such as dopamine agonists, benzodiazepines, opioids, and gabapentin;
5. A history of other motor disorders, such as Parkinson's disease, dyskinesia, and dystonia;
6. With an artificial cardiac pacemaker;
7. Pregnancy or breastfeeding.

#### 2.2.6 Randomization and allocation concealment

After initial assessments, subjects will be assigned to 1 of 2 groups with a 1:1 allocation ratio according to a computer-generated randomization list. The group each subject is allocated to will be concealed in sequentially numbered sealed opaque envelopes. The envelopes will only be opened after the subject has completed baseline clinical assessments. Randomization allocation will be concealed from the physician, subjects and evaluators.

### 2.3 Interventions

#### 2.3.1 Explanation for the choice of comparators

1. The period on placebo does not entail any additional risk of serious or irreversible harm to the patient.
2. The patient is capable of giving, and gives, fully informed consent.
3. The patient may request conventional treatment at any stage, or may be placed on such treatment by the treating physician.

#### 2.3.2 Intervention description experimental group: electroacupuncture

**Figure 2:**
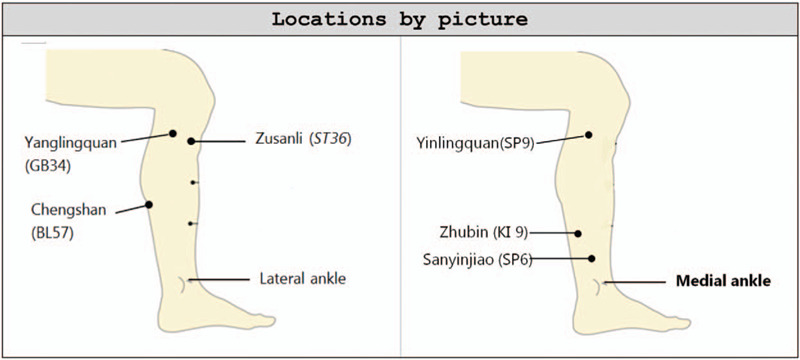
Locations of acupoints.

Subjects will receive 8 EA treatments. Treatments will be performed 2 times a week for 4 weeks. Based on the theory of Traditional Chinese Medicine (TCM) and clinical literature on acupuncture therapy for the obese, the acupoints are as follows: ST36 (Zusanli), GB34 (Yanglingquan), SP9 (Yinlingquan), SP6 (Sanyinjiao), BL57 (Chengshan) and KI9 (Zhubin). All acupoints are localized according to the WHO Standard Acupuncture Locations and exhibited in Table 1 and Fig. 2. Treatment will be performed by licensed acupuncturists who have at least 5 years of experience in acupuncture. The sterile disposable acupuncture needles (length of 40 mm, diameter of 0.27 mm) were developed by Tennyson Medical Instrument Developing Co., Ltd, Taiwan.

**Table 1:** Locations of acupoints in acupuncture group.

Acupoints	Locations
Zusanli (*ST36*)	3 cun^∗^ directly below Dubi (*ST35*), and one finger-breadth lateral to the anterior border of the tibia
Yanglingquan (GB34)	On the lateral aspect of the lower leg, in the depression anterior and inferior to the head of the fibula.
Yinlingquan (SP9)	On the medial aspect of the lower leg, in the depression of the lower border of the medial condyle of the tibia.
Sanyinjiao (SP6)	On the medial aspect of the lower leg, 3 cun above the medial malleolus, on the posterior border of the medial aspect of the tibia.
Chengshan (BL57)	On the posterior midline of the lower leg between UB 40 and UB 60. On the half way between the crease at the back of the knee and the ankle.
Zhubin (KI 9)	On the medial aspect of the lower leg, 5 cun directly above KID 3, on the line connecting KID 3 and KID 10, at the medial and inferior end of the belly of m. gastrocnemius.

∗ 1 cun (≈20 mm) is defined as the width of the interphalangeal joint of patients thumb.

The acupuncture points were stimulated by needle insertion, then manipulations of twirling, lifting, and thrusting performed on all needles for at least 30 seconds to reach De qi (a compositional sensation including soreness, numbness, distention, and heaviness). Among the 7 acupunctural points mentioned above, only ST36 (Zusanli), GB34 (Yanglingquan), SP9 (Yinlingquan), and KI9 (Zhubin) with electrical stimulation are conducted. The frequency of electrical stimulation is 2 Hz, and the intensities of the stimulation are below 9.8 mA for 15 minutes.^[16,17]^

#### 2.3.3 Control group: sham electroacupuncture

Although it remains methodologically challenging to establish an inert and concealable sham EA control, superficial insertion at non-acupuncture points plus no electrical stimulation is the most commonly used approach for administering sham treatments among EA trials on the basis of a literature review.^[18,19]^ Sham EA in this study is delivered by on non-acupuncture points plus superficial insertion plus no electrical stimulation.

All interventions shall be the same as those of the experimental group except the location of the acupoints. The sham acupoints shall be about 1.0 cm from the real acupoints in the experimental group and are spots that do not belong to standard acupoints on the body and are not suitable for actual acupuncture based on anatomical and other conditions, without needle manipulation for De qi. Besides, the dummy mode of the apparatus was used, in which it produced sound signals but no electrical current. All application will be performed by the same physician.

#### 2.3.4 Criteria for discontinuing or modifying allocated interventions

1. Electroacupuncture will be discontinued if the patients suffer from any adverse events (AEs).
2. The number of complications reported to the FDA is relatively low, given that millions of people receive acupuncture treatment each year. You have the right to withdraw from this study at any time during the research, which will not affect your rights and you will not be discriminated or retaliated against.

### 2.4 Outcomes

The primary outcome was the change in activity level of HRV in the RLS patients. The second outcome was the change in IRLSRS and ISI scores.

This study monitored physiological parameter changes after EA treatment. Blood pressures, heart rate (HR) and HRV were measured using an ANSWatch monitor developed by Taiwan Scientific Corp./ Ledron Technology Corp., Taiwan. The HRV is analyzed in both time and frequency domains to give rise to parameters that are linked to total ANS activity [HRV or SDNN (standard deviation of NN intervals); TP (Total Power)], sympathetic activity (LF %), parasympathetic (or vagal) activity (HF %), and sympathy-vagal balance (LF/HF) indexes.

The IRLS Rating Scale was developed by the International Restless Legs Syndrome Study Group (IRLSSG) to assess the severity of a patients RLS symptoms. Ten questions are listed in the questionnaire and scored from 4 (usually “very severe”) to 0 (usually none). The sum of the item scores is graded from 0 to 40. The scoring criteria are: Mild (score 1–10); Moderate (score 11–20); Severe (score 21–30); Very severe (score 31–40).^[20]^

The other questionnaire is ISI designed as a brief screening tool for insomnia. The 7-item questionnaire asks respondents to rate the nature and symptoms of their sleep problems using a Likert-type scale. The sum of the item scores is graded from 0 (no clinically significant insomnia) to 28 (severe).^[21]^

### 2.5 Follow-up

Follow-up observations will be conducted before the trials, during the treatment, and 1 month after the last treatment. The schedule of enrolment, intervention, and assessments is shown in Table 2.

**Table 2:** Schedule of enrollment, interventions, and assessments.

	Study period									
			Post-allocation							Close-out
Time-point	Enrolment	Allocation	T1	T2	T3	T4	T5	T6	T7	T8
Enrolment:										
Eligible screen	X									
ICF	X									
Allocation		X								
Intervention:										
Experimental group				X				X		
Control group				X				X		
Assessment:										
HRV recordings			X		X		X		X	X
IRLSRS			X		X		X		X	X
ISI			X		X		X		X	X
Adverse events				X	X		X	X	X	X

ICF:informed consent form. T1:before 1st intervention. T2:1st intervention, 8 treatments/4 weeks. T3:after 1st intervention. T4:a 2-week washout period. T5:before 2nd intervention. T6:2nd intervention, 8 treatments/4 weeks. T7:after 2nd intervention. T8:1 month after the last treatment.

### 2.6 Sample size

This study is a pilot study for evaluating the efficacy of EA compared to sham EA, and to evaluate the feasibility of large clinical trials. In the hopes of decreasing the subject drop-out rate, the trial is designed to only last 4 weeks. The minimum sample size for estimating the difference in the mean change in activity level of HRV between the experimental and control groups during the study period with an effect size of 0.54 (means of 3.3 and −3.7, standard deviations of 13.8 and 12.3, respectively; α = 0.05; 1–β = 0.8). Assuming a drop-out rate of 10%, the desired sample size for this pilot study is 112 subjects, with 56 in each group.

Based on the a priori calculation in the statistical software G∗Power 3.1.9.2, it was determined that a minimum total sample size of 124 would be needed in the independent-samples *t* test to show a significant difference.

### 2.7 Data collection and management

#### 2.7.1 Plans for assessment and collection of outcomes

All researchers including physicians, outcome assessors, and statisticians will receive training regarding data management. Data will be inputted into the medical record form of electronic computer which will be established before recruitment. The research assistant are responsible for collecting and verifying the accuracy of data.

### 2.8 Data management

The research assistant will keep tracking the direction (conditions) of post-treatment participants. Any personal information will remain confidential in a safe. Only the principal investigators can get the assessment.

### 2.9 Confidentiality

This study-related information will be stored securely at the study site. All participant information will be stored in locked file cabinets in areas with limited access. All laboratory specimens, reports, data collection, process, and administrative forms will be identified by a coded ID number only to maintain participant confidentiality. All records that contain names or other personal identifiers, such as locator forms and informed consent forms, will be stored separately from study records identified by code number. All local databases will be secured with password-protected access systems. Forms, lists, logbooks, appointment books, and any other listings that link participant ID numbers to other identifying information will be stored in a separate, locked file in an area with limited access.

### 2.10 Statistical methods

#### 2.10.1 Statistical methods for primary and secondary outcomes

Statistical analysis will be performed by using the software program Statistical Package for the Social Sciences (SPSS 22.0, IBM Corp., Armonk, NY). Participants demographic information and levels of major measured variables were analyzed by descriptive statistics. The clinical indices will be calculated, after which the evaluation between the 2 groups will be compared using Student *t* test for continuous data and Chi-Squared or Fisher exact test for categorical data. The difference of evaluation scores before and after each examination point will be analyzed using paired *t* tests.

A mixed model will be used to assess the quality of the cross-over design, wherein the dependent variable is the clinical index and the main independent variables are acupuncture type, administration order, and their interaction. After testing the interaction, if there is no significant carry-over effect in the interaction model and no significant order effect in the main effect model, it could be confirmed that the quality of the cross-design in this study is acceptable.

The generalized estimating equation (GEE) to fit a linear regression model will be used to analyze longitudinal data of the 2 acupuncture modes for each clinical index across the 4 data collection points (treatments for 8 weeks) that yields robust standard errors estimated in the model. A value of P &lt; 0.05 will be regarded as statistically significant for the above statistical analyses.

## 3 Discussion

Current drug treatments, like dopaminergic drugs, have been widely long term used for RLS. While some previous have showed an initial improvement in symptoms, longer studies, and clinical experience are reported that either treatment efficacy decreases with time and dopaminergic augmentation has been reported to be the major cause for treatment failure in RLS.^[22]^

Several lines of evidence suggest that patients with RLS have autonomic system abnormalities. Symptoms related to the autonomic nervous system have been more frequently reported in patients with RLS.^[23]^ Whereas Cikrikcioglu et al reported that elevated sympathetic activity might be beneficial in relieving RLS symptoms,^[24]^ Yildiz et al indicated that there is a relationship between RLS and increased sympathetic cardiac modulation.^[25]^ It shall pay high attention to understand the mechanisms of autonomic nervous system functions in hemodialysis patients with RLS

Traditional Chinese medicine has been shown to may have beneficial effects in RLS, for example, acupuncture. However, to our knowledge those trials were either non-randomized and had a limited number of subjects, or were subjective assessments. A washout period trial between treatment sessions was not used in previous randomized crossover studies of acupuncture on RLS.

Our study is the first investigation to analyze the change of HRV after EA in hemodialysis patients with RLS. We did this washout period to 2 weeks to further reduce potential carryover effects. The decision to use the SDNN, TP, LF, HF, LF/HF as the primary endpoint reflects the standard approach at the time the study was planned. In addition, an ANSWatch monitor provides an objective assessment of leg activity. The IRLSRS and ISI scores have often been used in evaluating RLS in previous studies;^[26,27]^ however, they are subjective assessments so we used them as secondary endpoints in this study.

We hypothesize that EA can help patients improve the abnormal leg activity in hemodialysis patients with RLS. If our hypothesis is correct, we may recommend subjects to receive EA treatment. In other words, EA appears to be an effective alternative in terms of cost and safety.

## 4 Trial status

IRB (protocol No.: Y_105_0300) was initially approved on August 23, 2019. NCT04356794 was registered on April 22, 2020. Recruitment began in May 2020. Expected date when recruitment will be completed on October 31, 2021.

## Acknowledgments

We appreciate the researchers, who have supported us with their helpful suggestions.

## Author contributions

JMC contributed to conception, design of the study, and manuscript writing.

PFC, YCC and LCL participated in the study conception and the analysis of the data. All authors read and approved the final manuscript.

**Data curation:** Jia-Ming Chen.

**Formal analysis:** Yu-Jun Chang, Po-Chi Hsu.

**Investigation:** Jia-Ming Chen.

**Methodology:** Jia-Ming Chen, Lun-Chien Lo.

**Project administration:** Jia-Ming Chen.

**Resources:** Jia-Ming Chen,Ping-Fang Chiu, Chia-Chu Chang.

**Software:** Yu-Jun Chang.

**Supervision:** Jia-Ming Chen, Lun-Chien Lo.

**Validation:** Lun-Chien Lo.

**Visualization:** Jia-Ming Chen, Lun-Chien Lo.

**Writing – original draft:** Jia-Ming Chen.

**Writing – review & editing:** Jia-Ming Chen, Lun-Chien Lo.

## References

[1] Rizzo V Aricò I Liotta G Impairment of sensory-motor integration in patients affected by RLS. J Neurol 2010;257:1979–85. 20635185 10.1007/s00415-010-5644-y

[2] Schlesinger I Erikh I Nassar M Restless legs syndrome in stroke patients. Sleep Med 2015;16:1006–10. 26116464 10.1016/j.sleep.2014.12.027

[3] Allen RP Earley CJ Restless legs syndrome: a review of clinical and pathophysiologic features. J Clin Neurophysiol 2001;18:128–47. 11435804 10.1097/00004691-200103000-00004

[4] Siddiqui S Kavanagh D Traynor J Risk factors for restless legs syndrome in dialysis patients. Nephron Clin Pract 2005;101:c155–60. 16020954 10.1159/000087073

[5] Mucsi I Molnar MZ Ambrus C Restless legs syndrome, insomnia and quality of life in patients on maintenance dialysis. Nephrol Dial Transplant 2005;20:571–7. 15671074 10.1093/ndt/gfh654

[6] Al-Jahdali HH Al-Qadhi WA Khogeer HA Restless legs syndrome in patients on dialysis. Saudi J Kidney Dis Transpl 2009;20:378–85. 19414938

[7] Kim J-M Kwon H-M Lim CS Restless legs syndrome in patients on hemodialysis: symptom severity and risk factors. J Clin Neurol 2008;4:153–7. 19513290 10.3988/jcn.2008.4.4.153 PMC2686851

[8] Merlino G Piani A Dolso P Sleep disorders in patients with end-stage renal disease undergoing dialysis therapy. Nephrol Dial Transplant 2006;21:184–90. 16144846 10.1093/ndt/gfi144

[9] La Manna G Pizza F Persici E Restless legs syndrome enhances cardiovascular risk and mortality in patients with end-stage kidney disease undergoing long-term haemodialysis treatment. Nephrol Dial Transplant 2011;26:1976–83. 21056943 10.1093/ndt/gfq681

[10] Billman GE The effect of heart rate on the heart rate variability response to autonomic interventions. Front Physiol 2013;4:222. 23986716 10.3389/fphys.2013.00222 PMC3752439

[11] Izzi F Placidi F Romigi A Is autonomic nervous system involved in restless legs syndrome during wakefulness? Sleep Med 2014;15:1392–7. 25266501 10.1016/j.sleep.2014.06.022

[12] Barone DA Ebben MR DeGrazia M Heart rate variability in restless legs syndrome and periodic limb movements of Sleep. Sleep Sci 2017;10:80–6. 28966745 10.5935/1984-0063.20170015 PMC5612042

[13] Pan W Wang M Li M Actigraph evaluation of acupuncture for treating restless legs syndrome. Evid Based Complement Alternat Med 2015;2015:343201. 25763089 10.1155/2015/343201 PMC4339862

[14] Lee J-H Kim K-H Hong J-W Comparison of electroacupuncture frequency-related effects on heart rate variability in healthy volunteers: a randomized clinical trial. J Acupunct Meridian Stud 2011;4:107–15. 21704953 10.1016/S2005-2901(11)60016-2

[15] Grant S Mayo-Wilson E Montgomery P CONSORT-SPI 2018 Explanation and Elaboration: guidance for reporting social and psychological intervention trials. Trials 2018;19:406. 30060763 10.1186/s13063-018-2735-z PMC6066913

[16] Heide AC Winkler T Helms HJ Effects of transcutaneous spinal direct current stimulation in idiopathic restless legs patients. Brain Stimul 2014;7:636–42. 25216650 10.1016/j.brs.2014.06.008

[17] Xu X-M Liu Y Jia S-Y Complementary and alternative therapies for restless legs syndrome: An evidence-based systematic review. Sleep Med Rev 2018;38:158–67. 28886918 10.1016/j.smrv.2017.06.003

[18] Chen Z-X Li Y Zhang X-G Sham electroacupuncture methods in randomized controlled trials. Sci Rep 2017;7:40837. 28106094 10.1038/srep40837 PMC5247761

[19] Jubb RW Tukmachi ES Jones PW A blinded randomised trial of acupuncture (manual and electroacupuncture) compared with a non-penetrating sham for the symptoms of osteoarthritis of the knee. Acupunct Med 2008;26:69–78. 18591906 10.1136/aim.26.2.69

[20] Walters AS LeBrocq C Dhar A Validation of the International Restless Legs Syndrome Study Group rating scale for restless legs syndrome. Sleep Med 2003;4:121–32. 14592342 10.1016/s1389-9457(02)00258-7

[21] Bastien CH Vallieres A Morin CM Validation of the Insomnia Severity Index as an outcome measure for insomnia research. Sleep Med 2001;2:297–307. 11438246 10.1016/s1389-9457(00)00065-4

[22] Garcia-Borreguero D Cano-Pumarega I Marulanda R Management of treatment failure in restless legs syndrome (Willis-Ekbom disease). Sleep Med Rev 2018;41:50–60. 29602660 10.1016/j.smrv.2018.01.001

[23] Shneyder N Adler CH Hentz JG Autonomic complaints in patients with restless legs syndrome. Sleep Med 2013;14:1413–6. 24152795 10.1016/j.sleep.2013.08.781 PMC4105217

[24] Cikrikcioglu MA Hursitoglu M Erkal H Oxidative stress and autonomic nervous system functions in restless legs syndrome. Eur J Clin Invest 2011;41:734–42. 21250984 10.1111/j.1365-2362.2010.02461.x

[25] Yildiz A Yildiz C Karakurt A Assessment of cardiac autonomic functions by heart rate variability in patients with restless leg syndrome. Turk Kardiyol Dern Ars 2018;46:191–6. 29664425 10.5543/tkda.2018.33896

[26] Trenkwalder C Hening WA Montagna P Treatment of restless legs syndrome: an evidence-based review and implications for clinical practice. Mov Disord 2008;23:2267–302. 18925578 10.1002/mds.22254

[27] Carrier J Frenette S Montplaisir J Effects of periodic leg movements during sleep in middle-aged subjects without sleep complaints. Mov Disord 2005;20:1127–32. 15884036 10.1002/mds.20506

